# Ultra-low Concentration of Cellulose Nanofibers (CNFs) for Enhanced Nucleation and Yield of ZnO Nanoparticles

**DOI:** 10.1021/acs.langmuir.2c01713

**Published:** 2022-10-06

**Authors:** Billy W. Hoogendoorn, Björn K. Birdsong, Antonio J. Capezza, Valter Ström, Yuanyuan Li, Xiong Xiao, Richard T. Olsson

**Affiliations:** † Department of Fibre and Polymer Technology, School of Engineering Sciences in Chemistry, Biotechnology and Health, KTH Royal Institute of Technology, Teknikringen 56, 114 28Stockholm, Sweden; ‡ Department of Material Science and Engineering, School of Industrial Engineering and Management, KTH Royal Institute of Technology, Brinellvägen 23, SE-100 24Stockholm, Sweden

## Abstract

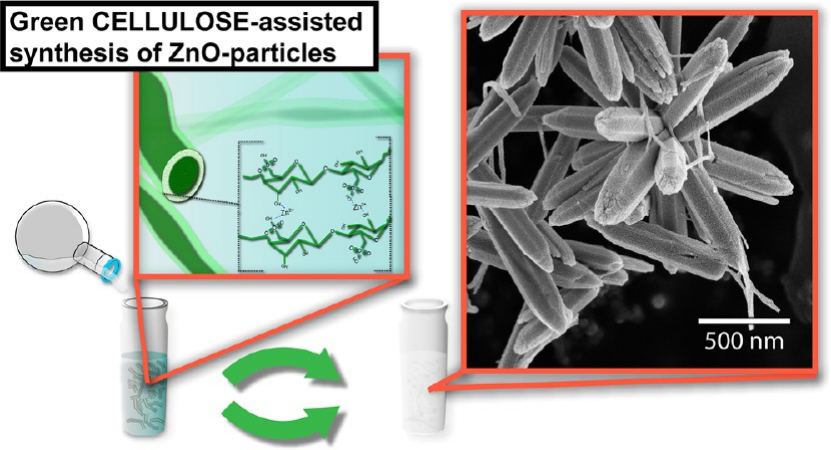

Cellulose nanofibers (CNFs) were used in aqueous synthesis protocols for zinc oxide (ZnO) to affect the formation of the ZnO particles. Different concentrations of CNFs were evaluated in two different synthesis protocols producing distinctly different ZnO morphologies (flowers and sea urchins) as either dominantly oxygen- or zinc-terminated particles. The CNF effects on the ZnO formation were investigated by implementing a heat-treatment method at 400 °C that fully removed the cellulose material without affecting the ZnO particles made in the presence of CNFs. The inorganic phase formations were monitored by extracting samples during the enforced precipitations to observe changes in the ZnO morphologies. A decrease in the size of the ZnO particles could be observed for all synthesis protocols, already occurring at small additions of CNFs. At as low as 0.1 g/L CNFs, the particle size decreased by 50% for the flower-shaped particles and 45% for the sea-urchin-shaped particles. The formation of smaller particles was accompanied by increased yield by 13 and 15% due to the CNFs’ ability to enhance the nucleation, resulting in greater mass of ZnO divided among a larger number of particles. The enhanced nucleation could also be verified as useful for preventing secondary morphologies from forming, which grew on the firstly precipitated particles. The suppression of secondary growths' was due to the more rapid inorganic phase formation during the early phases of the reactions and the faster consumption of dissolved salts, leaving smaller amounts of metal salts present at later stages of the reactions. The findings show that using cellulose to guide inorganic nanoparticle growth can be predicted as an emerging field in the preparation of functional inorganic micro/nanoparticles. The observations are highly relevant in any industrial setting for the large-scale and resource-efficient production of ZnO.

## Introduction

Nanocellulose has been acknowledged for its extraordinary mechanical properties along the fibers (*ca.* 7 GPa) in relation to its low density (1500 kg/m^3^),^1−4^ extensive specific surface area when defibrillated (reaching >100 m^2^/g^5^), and tuneable surface characteristics.^6−8^ Nanocellulose has also been shown to interact with metal ions,^9−11^ which is a feature that has been highlighted in the context of associating metal ions to cellulose for the removal of heavy metal ions.^12−14^ The cellulose/metal ion interactions have however been scarcely studied when metal ions are precipitated into a solid phase in the presence of cellulose, although some examples of cellulose’s ability to function as a template for grafting metal oxides do exist.^15,16^ Even smaller attention has been given to cellulose in the context of affecting the intrinsic structural features of the precipitated inorganic phase (crystal phases, crystallinity, external crystal surfaces) and/or particle morphologies and sizes. This is rather surprising since cellulose is the most abundant natural polymer on earth.^4^ Metal oxide micro/nanoparticles are at the same time strongly dependent on particle size and morphologies as well as the crystal nature and surface termination of the metal oxide in any functional context ranging from photovoltaics to energy storage systems.^17−22^

A benefit with water-based preparation methods of metal oxide micro/nanoparticles is the environment-friendly and mild conditions used where cellulose can be readily dispersed.^23,24^ The interactions between the cellulose surfaces and metal ion species can here allow for using nanocellulose as a tool to tune the morphology during the enforced precipitation of metal oxide particles. An interesting aspect is that cellulose nanofibers represent a category of materials that contains a vast diversity of associated properties depending on its peparation conditions, which provides versatility in affecting the precipitation conditions. On top of the natural variations that can be associated with the source of the cellulose, it is well established that facile and inexpensive reactions can be used for modifying the surface of the cellulose,^6−8^ making the nanofibers possibly one of the most inexpensive additives that can be used in the preparation of functional metal oxides for applied material science. Cellulose nanofibers (CNF) can be prepared on a large scale from a variety of sources, including wood and plants as well as bacterial and marine resources.^4,25^

In this work, the effect nanocellulose (at concentrations 0.05–0.1 wt %) has on the formation of zinc oxide (ZnO) in the aqueous precipitation of nanostructured micron-sized particles is reported. ZnO is characterized by the highly anisotropic characteristics of the wurtzite crystal lattice, which has been widely documented to significantly affect its functional properties.^21,22^ Two different synthesis protocols resulting in ZnO morphologies with a different domination of O- and Znterminations were studied: one producing an oxygen-rich sheet composed of flower-shaped particles and one producing predominantly zinc-terminated rod-composed sea-urchin-shaped particles.^21^ It is demonstrated that the introduction of CNF in an aqueous reaction of ZnO resulted in decreased particle sizes of up to 50% when making flower-shaped particles, and a similar decrease of 45% was observed when producing sea-urchin-shaped particles. The effect on particle sizes was at the same time accompanied by increases in the total yields of the reactions with 13 and 15%, respectively. Overall, the study shows that very small amounts (<0.5 wt %) of highly crystalline cellulose nanofibers, as a renewable resource material, significantly influenced the preparation of the inorganic ZnO particles with differently tailored surfaces (zinc or oxygen terminated).

## Experimental Section

### Materials

Bacterial cellulose (BC) grown from strains of *Acetobacter xylinum* bacteria was purchased as preserved in saccharide solutions from Monstra LLC (dba Pacific Rim), Thailand. Reagent grade sulfuric acid (95–97%) and sodium hydroxide (≥98%) were purchased from Sigma-Aldrich. Zinc nitrate hexahydrate, Zn(NO_3_)_2_·6H_2_O (98%), was purchased from Fischer Scientific. MilliQ water (MQw), 18.2 MΩ, 25 ^°^C, pH 7.0, was used in all procedures.

### Preparation of Cellulose Nanofibers (CNFs) from Bacterial Cellulose (BC)

The protocol for preparing the bacterial CNFs was derived from a previously reported method of acid hydrolysis of bacterial cellulose.^26^ Two kilograms of preserved BC was refined by water-phase exchange every 12 h for 48 h, eliminating the preservative sugar and residual growth medium. The BC was then washed for 2 × 20 min in 2 L of boiling 10 vol % NaOH solution to remove all organic material remaining from the bacterial biosynthesis. The BC was neutralized by exchanging the water phase until a pH = 7 was reached. The BC was then shredded (in a Blendtec 625 blender) before being compressed into a compact and wet fibrous mass. The procedure resulted in 68 g of wet bacterial cellulose with a solid content of *ca.* 10 wt % dry fibers (established from oven drying at 70 °C).

The extraction of crystalline cellulose nanofibers was made by adding 34 g of the compressed bacterial cellulose, within a time frame of 3 min, into 500 mL of 30 vol % H_2_SO_4_ (aq) held at 80 ± 1 °C. The extraction proceeded under mechanical stirring (200 rpm) for 9 h before being quenched by the addition of 500 mL of 10 °C MQw. The mixture was centrifuged three times for 10 min at 11,000*g* (15 °C) using a Sorvall RC-5B Plus centrifuge. The supernatant was exchanged with MQw between the cycles, and the cellulose was redispersed using a high shear mixer (IKA T25 Digital Ultra Turrax) for 5 min at a speed of 15,000 rpm. The final CNF dispersion (master dispersion) had a solid content of 0.4 ±0.02 wt%. Figure S1 shows the XRD diffractogram of the refined CNF, with a micrograph displaying the length and width of the nanofibers determined to be 575 ± 30 and *ca.* 20 nm, respectively. The average values were established from a minimum of 1000 measurements using ImageJ (National Institute of Health, Maryland, USA). The master dispersion was diluted to concentrations useful in the mixing with the metal salts referred to in Table 1. During the dilution, the pH was adjusted to a value of 7 by using a 5 mM NaOH solution (aq).

**Table 1:** Concentrations of the Zinc Salt Solutions (*C*_Zn(NO_3_)26(H_2_O)_), Cellulose Dispersions (*C*_CNF-disp_), and NaOH Solutions (*C*_NaOH_), Respectively, for Each Reaction^a^

sample order	sample specification	*C*_Zn(NO_3_)__2__6(H_2_O)_ (M)	*C*_CNF-disp_ (g/L)	*C*_NaOH_ (M)	*C*_CNF_ (g/L)^b^	*T* (°C)
run 1	ZnO_Ref_-flower	0.45	0	3	0	40
run 2	ZnO_CNF1_-flower	0.45	0.1	3	0.05	40
run 3	ZnO_CNF2_-flower	0.45	0.2	3	0.1	40
run 4	ZnO_Ref_-sea-urchin	0.45	0	4.5	0	60
run 5	ZnO_CNF1_-sea-urchin	0.45	0.1	4.5	0.05	60
run 6	ZnO_CNF2_-sea-urchin	0.45	0.2	4.5	0.1	60

a CNF1 and CNF2 refer to the final cellulose concentrations of 0.05 and 0.1 g/L in the mixed solutions.

b *C*_CNF-disp_ is the concentration of CNF in the CNF dispersion prior to mixing it with the zinc salt solution, while *C*_CNF_ is the concentration of CNF after mixing all constituting dispersions and solutions together.

The surface charge was determined on the neutralized CNF dispersion by performing polyelectrolyte titration using a Stabino Particle Charge unit (ParticleMetrix GmbH, Germany). Briefly, 0.5 mL of the CNF dispersion was diluted to 10 mL prior to the measurements using MQw, resulting in a concentration of 0.04 wt %. The titration was performed using a polydiallyldimethylammonium chloride (p-DADMAC, Fujifilm Wako Chemicals) specifically developed for colloidal titration. The purchased commercial solution with a charge of 0.0025 mol/mL was diluted until a charge of 0.3045 μmol/mL was obtained, which according to the manufacturer of the instrument was optimal for carrying out the measurements. The CNF dispersion was titrated with the diluted P-DADMAC solution at a rate of *ca.* 0.004 mL/s, while under stirring, until the equivalence point of the measured zeta potential was reached. The addition of sodium ions to the CNF dispersion (prior to titration) had an insignificant effect on the titration (estimated to be <10^–8^ mol/L). After determining the charge of four different samples (replicates), the average surface charge was determined to be 80 ±20 μeq/g (pH = 7.0 ± 0.1). The protocols used were the same as previously reported protocols using the above instrument.^27,28^

### Preparation of ZnO Particles

After mixing the constituents according to Table 1, the reaction mixtures contained 0, 0.05, or 0.1 g/L of CNFs. The precipitation conditions were adopted from previous articles, targeting conditions that generated morphologies reported as “flower-shaped particles” dominated by atomic oxygen terminations^29,30^ or “sea-urchin-shaped particles”^23,30,31^ dominated by Zn terminations in the rod-like structures embedded in the sea urchin morphologies.^23^ The zinc nitrate solutions (250 mL) were mixed into the CNF dispersion (500 mL) by adding the zinc nitrate into the CNF dispersion under vigorous stirring. The solutions were then stirred (350 rpm) at ambient conditions overnight (23 °C), after which the pH had stabilized at ∼4. For the low concentrations used as a nucleation support, the pH remained the same after the overnight stirring. The CNF/metal-ion solutions were transferred to a cylindrical plastic reactor in a tempered oil bath, heated to 40 or 60 °C (Table 1) with stirring, for 3 h.

The precipitation reaction was initiated by adding 250 mL of the NaOH solution (Table 1) that had been separately preheated to the same temperature as the CNF/Zn-ion mixture with 3 s addition time. The reaction progressed for 1 h at the specified temperatures before being quenched by the addition of 700 mL of MQw. The quenched mixture, containing a white precipitate, was centrifuged for 4 ×20 min at 11,000*g*, with the supernatant being exchanged with pure MQw between each cycle. During each 1 h reaction, 4 mL aliquots were taken after 1, 15, 30, and 60 min. The samples were immediately quenched in an ice bath and centrifuged for 4 × 2 min at 12,100*g* with an intermediate change of the supernatant. The reproducibility of the reactions was established by triplicate reactions.

### Postsynthesis Treatment of ZnO/Cellulose Hybrid Materials

**Figure 1:**
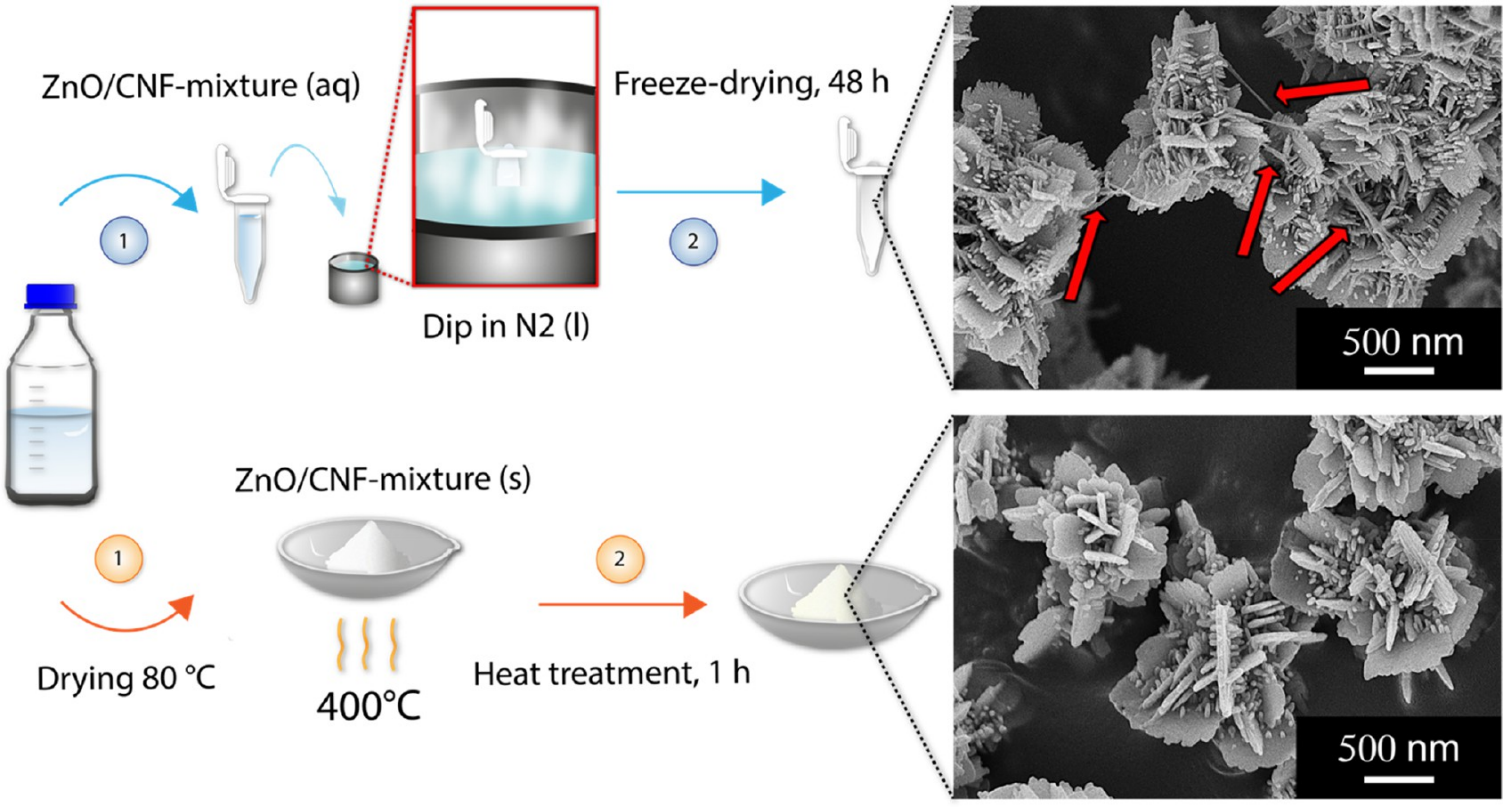
Schematic illustrating the post-treatment procedures with the removal of the CNF fraction through heat treatment demonstrated at the bottom and the freeze-drying procedure of the ZnO/CNF hybrid material at the top. The micrographs represent the flower-shaped ZnO particles produced in the presence of 0.1 g/L of CNFs after the heat-treatment (bottom) and freeze-drying (top), respectively, with the arrows highlighting the remaining CNFs.

The ZnO/CNF materials were prepared for characterization using two different methods. In the first method, the ZnO/CNF material dispersions were frozen quickly using liquid nitrogen. Dispersions were freeze dried for 48 h, preserving the frozen structure of the ZnO/CNF hybrid material. In the second method, the ZnO/CNF material was dried in an oven at 80 °C (until no change in mass was detected, ensuring the complete removal of water). The dried material was then heat-treated in a furnace at 400 °C for 1 h, resulting in the complete degradation and removal of the cellulose without affecting the morphology of the ZnO phase.^21,23^ The micrographs in Figure 1 show the ZnO flowers produced after heat treatment at 400 °C (bottom) and freeze-drying (top). The removal of CNF was confirmed for the samples exposed to 400 °C, which was in contrast to the freeze-dried material wherein the cellulose remained as highlighted by the arrows in the micrograph. The micrographs additionally confirmed that the morphology remained the same regardless of the heat-treatment procedure.

### Characterization of ZnO and ZnO/BC Hybrids

#### Mass/Yield

The amount of formed inorganic material (particles) was determined by drying the obtained product from each reaction at 80 °C until no decrease in mass was detected and thereafter subtracting the known mass of added CNF. The yield was determined from the ratio of the mass of the inorganic material and the theoretical mass that would result from the complete conversion of metal salts to ZnO (see eq 1).
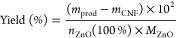
1

In eq 1, *m*_prod_ is the weighed dry mass obtained after the respective reactions, *m*_CNF_ is the known mass of CNF prior to the reaction, *n*_ZnO_(100%) is the amount of moles of ZnO obtained assuming a full conversion from Zn^2+^ to ZnO, and *M*_ZnO_ is the molar mass of ZnO.

#### Microscopy

A Hitachi S-4800 field emission scanning electron microscope (FE-SEM) was used. The samples were placed on a conductive carbon tape before being coated with a Pt/Pd coating for 40 s at a current of 80 mA using a Cressington HR sputter coater (model 208RH). The images were taken at an acceleration voltage of 5 kV and an emission current of 10 μA. The micrographs were used to determine the sizes of the formed particles by measuring the diameters of exactly 600 particles per sample using ImageJ.

#### X-ray Diffraction (XRD)

XRD was performed on freeze-dried samples of the CNF, the freeze-dried CNF/zinc nitrate solution, and the material obtained in all of the 60 min reactions using a PANalytical X’Pert PRO X-ray diffractometer. The measurements were performed at 45 kV and 40 mA (with Cu Kα radiation) using a starting angle of 5° and a stop angle of 80°. The step size was 0.06° with a wavelength of 0.154 nm. The Scherrer equation (eq 2)^32^ was used to estimate the crystallite size, where *k* is a shape factor, λ is the X-ray wavelength (nm), β is the width of the peak at the half-maximum intensity of the diffraction peak (rad), and θ is the Bragg angle (rad).
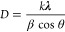
2

Equation 3 was used to calculate the lattice parameters *a* and *c*.^33^
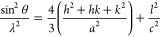
3where *h*, *k*, and *l* in eq 3 stand for the Miller indices.

The crystallinity of the bacterial CNF was determined to be 80–90% by using the “peak deconvolution method” where Gaussian curve-fitting is performed to separate the crystalline contribution of the peak from the amorphous halo. The crystallinity was then determined using eq 4.^34^
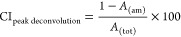
4where *A*_(am)_ is the area of the amorphous halo and *A*_(tot)_ is the total area of the crystalline peaks.

#### Specific Surface Area (SSA)

The surface area of ZnO was determined by Brunauer–Emmett–Teller (BET) absorption/desorption tests of nitrogen on the ZnO particles (Micromeritics ASAP 2020).

Inductively coupled plasma-optical emission spectrometry (ICP-OES) was used to study the association of zinc ions to CNF prior to the reaction. Zn-ion/CNF mixtures were prepared with a CNF concentration of 3 g/L and a zinc nitrate hexahydrate concentration of 0.11 M (similar Zn-ionic concentration as the reaction mixtures prior to initiating the synthesis). The zinc salt and CNF were mixed together in a similar way as described in the Experimental Section, with a zinc nitrate hexahydrate dispersion being mixed with a CNF dispersion. The zinc-ion/CNF mixture was stirred overnight at ambient conditions before being conditioned at 60 °C for 3 h (in exactly the same fashion as for the reactions). The CNF was separated from the aqueous supernatant *via* centrifugation. The metal ion content and concentrations within the two fractions were determined by performing elemental analysis on the supernatant and compared to concentration of the zinc nitrate solution used when preparing the Zn-ion/CNF mixture. The concentrations of zinc were established by ICP-OES (Thermo Fisher iCAP 7400, USA).

#### Fourier Transform Infrared Spectroscopy (FTIR)

The infrared spectra were measured on freeze-dried samples from all reactions as well as the acid hydrolyzed CNF and the CNF/zinc nitrate solution using a PerkinElmer Spectrum 100 instrument equipped with an ATR accessory and an MIR TGS detector. The measurements were performed at a scanning rate of 1 cm^–1^ at a resolution of 4 cm^–1^. The absorbance was measured 16 consecutive times between 600 and 4000 cm^–1^.

## Results and Discussion

### The Effect of CNFs on the ZnO Particle Size

**Figure 2:**
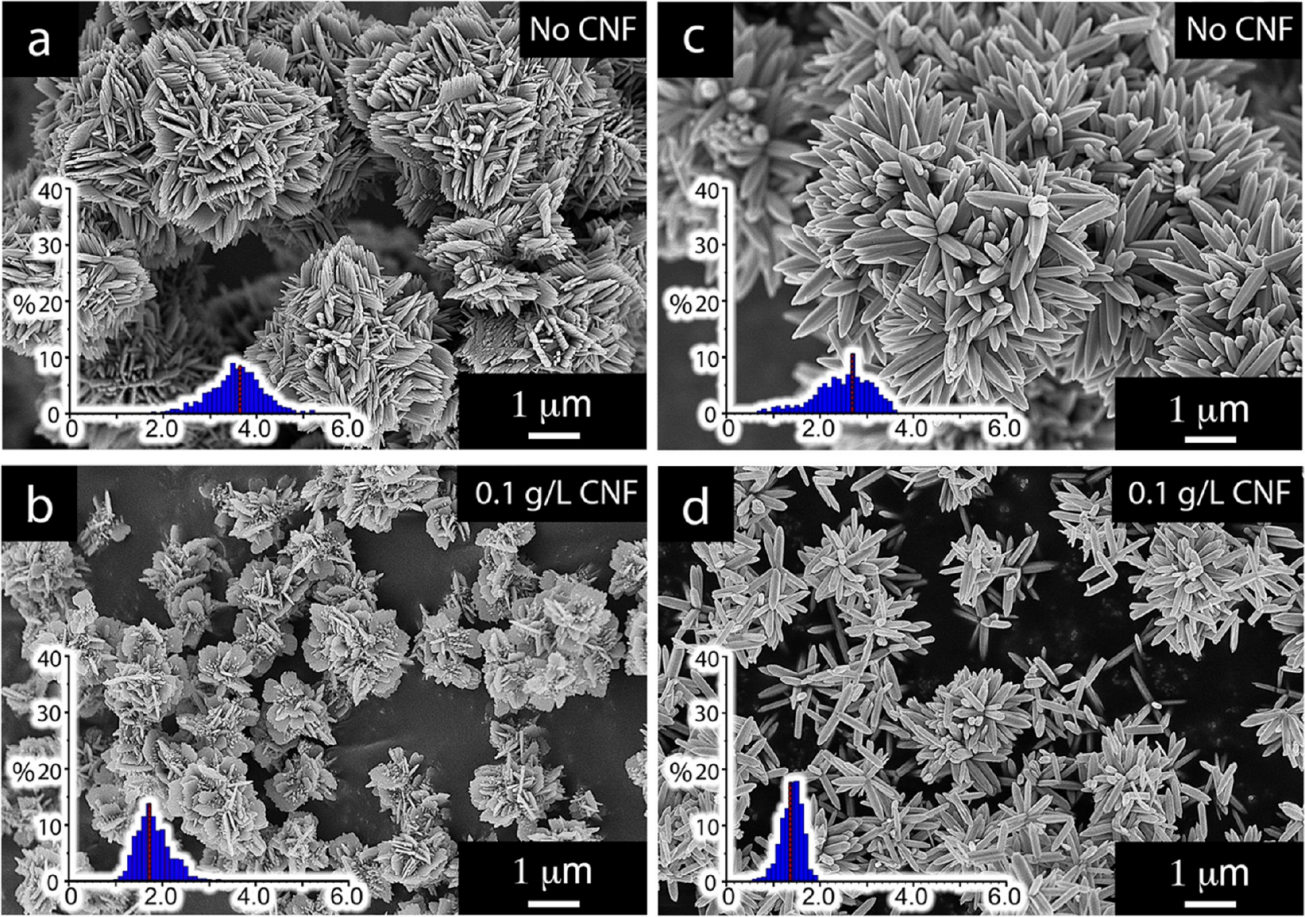
Micrographs of ZnO particles referred to as flowers a and b (run 1 and run 3 (Table 1)) and sea urchins c and d (run 4 and run 6 (Table 1)) after calcination at 400 °C. The effect of performing the synthesis in the presence of 0.1 g/L of CNF is demonstrated. The histograms show the distribution of particle diameters in micrometers for the flower-shaped particles (a: average size 3.52 μm and b: average size 1.76 μm) and the sea urchin particles (c: average size 2.45 μm and d: average size 1.34 μm) consisting of nanosheets and hexagonally faceted rods, respectively. The red lines represent the average particle sizes. All particles (regardless if synthesized with CNF) were calcinated for accurate comparison.

Figure 2 illustrates the effect of cellulose nanofibers (CNFs) at a concentration of 0.1 g/L on the aqueous formation of micron-sized zinc oxide particles. The reaction conditions are displayed in Table 1. The size of particles decreased by 50 and 45% for the flower particles (Figure 2b) and sea urchin particles (Figure 2d), respectively. At the same time, the nanometric substructure morphologies of the micron-sized particles changed, although the overall particle shapes remained the same. The larger *ca.* 3.5 μm flower particles (Figure 2a) built up by a mix of nanorods and nanosheets developed into *ca.* 1.8 μm particles (Figure 2b) entirely based on sheet morphologies. In the case of the *ca.* 2.5 μm sea urchin particles (Figure 2c), the only morphological change was a decrease in the rod length and a less defined hexagonally faceted substructure associated with the wurtzite ZnO rods (Figure 2d). A CNF concentration of 0.05 g/L further supported the findings of a decreasing particle size with the presence of CNF during the synthesis. As little as 0.05 g/L resulted in a decrease in average particle diameter to 2.6 μm (*ca.* 25%) for the flower structures and to 1.4 μm (*ca.* 41%) for the sea urchins. The reduced sizes of the ZnO particles occurred simultaneously with increased yields from 81 to 95% for the flower-shaped particles and 63 to 74% for the sea urchin particles. It is noteworthy that the reproducibility of the batches synthesized in the absence of cellulose showed higher deviations from batch to batch (±3%) compared to the CNF-containing batches, which showed a standard deviation of ±1.5%. The combination of increased reaction yields and smaller particle sizes accordingly in both cases resulted in an increased total surface area of the ZnO phase due to larger specific surface areas of the respective particles (Table 2) in combination with the greater mass of particles formed. It has previously been suggested that cellulose functions as a nucleating agent through its ability to interact with zinc ionic species,^35−37^ particularly by interacting either with the hydroxyl groups naturally present on the cellulose surface^38,39^ or with other charged functional groups added to the cellulose surface, such as carboxyl groups,^36^ thereby creating sites on which a ZnO phase can form. The effect on the reaction yield was not highlighted in these studies.

**Table 2:** Yield, Average Particle Diameter (*D*_particle_) for the Flower Structures/Average Rod Length (*L*_rod_) for the Sea Urchin Structures, and Specific Surface Area (SSA), Measured through BET, of the Flower-Shaped Particles and Sea-Urchin-Shaped Particles, Respectively^a^

flowers	yield (%)	*D*_particle_ (μm)	SSA (m^2^/g)	sea urchins	yield (%)	*D*_particle_ (μm)	SSA (m^2^/g)
ZnO_Ref_-flower (run 1)	81	3.52	5.45	ZnO_Ref_-sea-urchin (run 4)	63	2.45	1.98
ZnO_CNF1_-flower (run 2)	89	2.63	6.77	ZnO_CNF1_-sea-urchin (run 5)	73	1.44	5.03
ZnO_CNF2_-flower (run 3)	95	1.76	8.84	ZnO_CNF2_-sea-urchin (run 6)	74	1.34	5.59

a All yield values for the reactions obtained with CNF present were associated with standard deviations of approx. ±1.5%. Runs 1 and 4 (see Table 1) represent the reference reactions of the flower- and the sea-urchin-shaped particles, respectively, in the absence of cellulose (CNF) during the synthesis. Runs 2 and 5 (see Table 1) show the effect of introducing 0.05 g/L of CNF in the synthesis reactions, whereas runs 3 and 6 (see Table 1) show the results of the same synthesis reactions containing 0.1 g/L of CNF.

The presence of the CNF as a nucleation support allowed for a more dominant nucleation but also a precipitation (growth) over a shorter timespan (min), eliminating the formation of secondary morphologies on top of the primary structures. This sort of cellulose-induced nucleation has previously been observed for silver and iron oxide particles on the cellulose nanofibers using microscopy (TEM).^15,40,41^ The higher solubility of zinc ion species makes it challenging to demonstrate the zinc hydroxide complexes due to their limited thermodynamic stability at neutral pH, *i.e*., before the enforced precipitation at elevated pH (pH > 8).^42^ A possible prenucleation of the Zn-ionic species during the initial stages of the reaction scheme (prior to the enforced precipitation by adding the alkaline solution) was therefore suggested as an explanation for the markedly higher yields and smaller particle sizes. CNF concentrations of 1.5 and 1.8 g/L were therefore evaluated to investigate if increasing amounts of the CNF could contribute to forming even higher amounts (yields) of ZnO particles. The results showed that, for these systems, no further decrease in particle size or increase in reaction yields occurred (see Supporting Information Figure S5). This suggests that the active sites responsible for initiating the particle growth on the surface were already in excess at 0.1 g/L of CNF. The surface area of the CNF was previously estimated to be 159 m^2^/g.^43^

To verify the favorable interactions between Zn-ion species and CNF, the conditions before the enforced precipitation were replicated. After separating the cellulosic material from the aqueous medium through centrifugation and comparing the concentration in the supernatant to the total concentration of the solution through ICP measurements, it was revealed that the amount of Zn-ions concentrated to the vicinity of the cellulose nanofibers was shown to be 12.4% higher than the zinc-ion concentration in the supernatant (see Table S3). For the dried zinc nitrate solution exposed CNF, FTIR showed increases in peak intensities at 1630–1640, 1420–1430, and 1310–1320 as well as a bulging of the peak at 3340–3350 cm^–1^; see Figure S2. The changed appearance of the 1630–1640 peak and the 3340–3350 peak (both connected to interactions involving the OH groups) is suggested to be a consequence of the bridging/complexation between hydroxyl functional groups and the zinc ionic species.^44,45^

**Figure 3:**
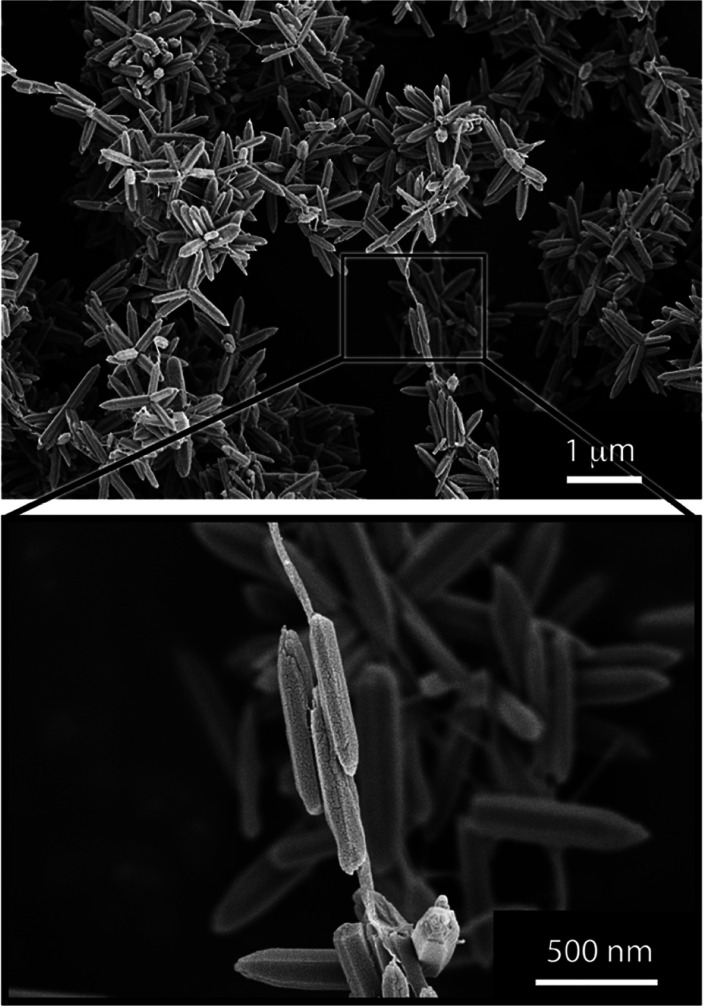
Micrographs of the freeze-dried samples from the reaction producing dominantly rod-based sea urchin structures in a CNF concentration of 0.1 g/L (see Table 1).

These recorded associations were however not sufficient to cause the zinc oxide particles to merge and unify with the cellulose fibers, as previously shown for iron oxide particles.^15,16^ The micrographs of the freeze-dried samples in Figure 3 show the ZnO rods as associated along the CNF surface and the relatively small amount of cellulose needed to affect the reactions. However, in contrast to previous work demonstrating the inorganic particles (CoFe_3_O_4_) occasionally entirely enclosing the fibers as embedded, the nucleated and grown ZnO could not be verified to integrate the fibers within the ZnO phase.^16^

### The Effect of CNF on the ZnO Phase Composition

**Figure 4:**
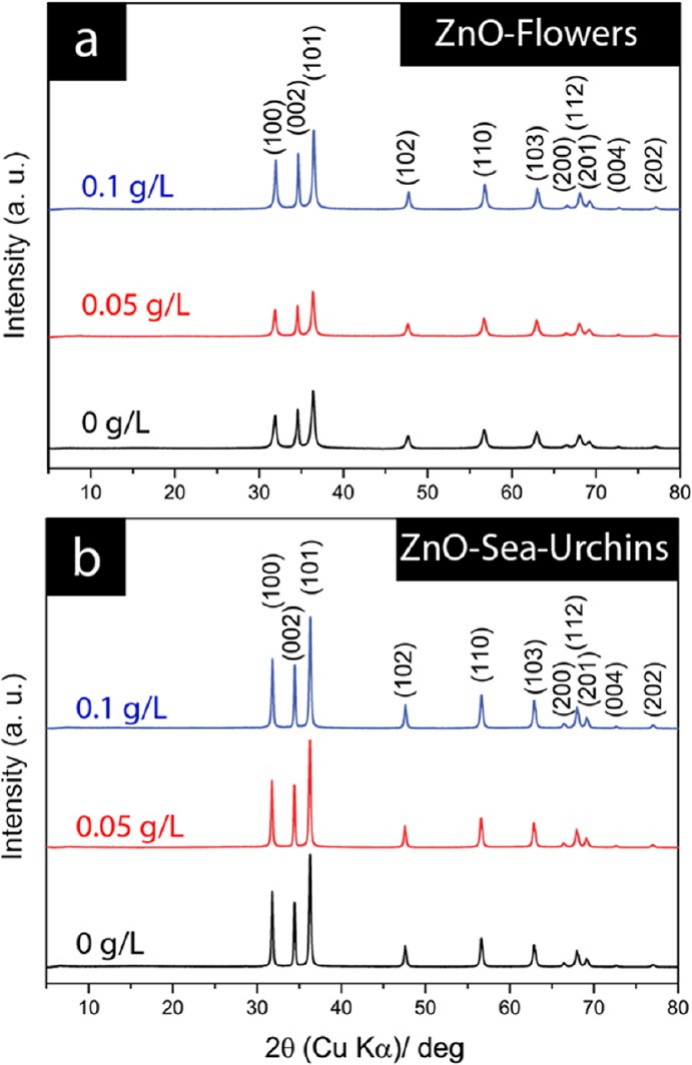
X-ray diffractograms of freeze-dried samples of the (a) flower particles and (b) sea urchin particles at CNF contents of 0 to 0.1 g/L. All diffraction peaks solely correspond to the ZnO wurtzite phase.

X-ray diffraction (XRD) was performed on freeze-dried samples from all reactions; see Figure 4. The diffractograms confirmed the crystal structure of the hexagonal wurtzite structure of ZnO (ICSD PDF 075-0576). The lattice parameters were determined to be *a* = 3.2 and *c* = 5.2 Å for the unit cell, with a pure ZnO phase and all reactions only providing the zinc oxide, regardless of the presence of the CNF. The bacterial CNF was not apparent in the diffractograms due to the small amounts in the samples (the presence of CNF could however be confirmed by FTIR spectroscopy; see Figure S2).

**Figure 5:**
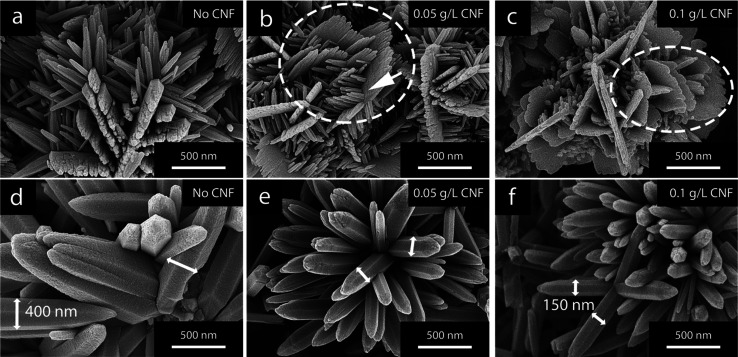
Micrographs of ZnO particles referred to as (a–c) flower-shaped and (d–f) sea-urchin-shaped. The effect of different amounts of CNF, 0.05 and 0.1 g/L, present during the ZnO-synthesis is shown for (b, c) flower structures and (e, f) sea urchin structures. The CNF was removed *via* thermal degradation at 400 °C before microscopy imaging.

Table 3 shows that the presence of the cellulose affected the crystallite size in the case of the flower-shaped particles, increasing from 22.7 to 27.6 and 32.3 nm, when the reaction was performed with an increasing amount of cellulose from 0 to 0.05 and further to 0.1 g/L CNF, respectively. The change in crystallite size was insignificant for the sea urchin particles consisting of spiky rods with a decreasing rod size as the CNF concentration increased; see Figure 5. The crystallite size always remained within 37 ±0.6 nm for the sea urchin particles regardless of the presence of CNF during the synthesis.

**Table 3:** Estimated Crystallite Sizes Using the Scherrer Equation for the (002) Peak for Each Respective Reaction

flower samples	crystallite size (nm)	sea urchin samples	crystallite size (nm)
ZnO_Ref_-flower	22.7	ZnO_Ref_-sea-urchin	36.3
ZnO_CNF1_-flower	27.6	ZnO_CNF1_-sea-urchin	37.3
ZnO_CNF2_-flower	32.3	ZnO_CNF2_-sea-urchin	37.5

With consideration to the morphological changes visible in Figure 5, it could be interpreted that the development of larger sheet morphologies, associated with the flower-shaped particles, was consistent with possibilities to house growth of larger in-plane crystallite sizes. For comparison, this orientational in-plane crystallite growth was never affected (compromised/constrained or enhanced) in the case of the rod-shaped morphologies, wherein the (002) plane was contained within the rods and apart from the main rod growth direction. The width of the rods was however significantly greater in size (>150 nm) than the crystallites; see Figure 5f.

Overall, the effects on crystallite revealed that the presence of CNF not only affected the size of the ZnO structures on a micron scale but also had an effect on the sizes of the crystalline domains.

### The Effect of CNF on Primary and Secondary Nucleation in the Growth of ZnO Morphologies

Figure 5 shows the ZnO morphologies with increasing content of CNF for the flower-shaped and sea-urchin-shaped particles. For the flower-shaped particles, an increasing cellulose content was synonymous with a stronger formation of sheet-like morphologies; see Figure 5c. For comparison, when no CNF was present in the reactions, the formation of sheet-like morphologies was disfavored during the preparation of flower-shaped particles (Figure 5a). The circle in Figure 5b highlights the formation of additionally grown smaller nanorods (see arrow), which started their growth on the surface of the sheet-like primary structures. The presence of these secondary structures extending on top of the sheet morphologies and the fact that their growth was initiated at later stages were synonymous with a strongly heterogeneous nucleation. For the rod-based sea-urchin structures, Figure 5d–f shows that the only effect the CNF had concerned the size of the individual rods in the sea urchins.

In addition to the decreased rod length (demonstrated in Figure 2c,d), a significant decrease in rod thicknesses was evident even at CNF concentrations as low as 0.05 g/L (see arrows in Figure 5), and the average rod thickness was reduced from *ca.* 400 nm to *ca.* 150 nm. For the sea urchins, secondary structures (originating from a strongly heterogeneous nucleation) on top of clearly defined primary structures (the rods) were never observed.

Zhang *et al*. demonstrated flower particles consisting of sheet-like morphologies (as in Figure 5) developed through a hydrothermal process carried out at elevated temperatures (30–100 °C).^29^ The formation of secondary structures on top of formed primary structures was achieved by adjusting the concentration of the precursor salt in the reactions.^29^ According to Zhang *et al.*, secondary structures could be prevented by limiting the concentration of Zn-ions. Research by Cho *et al.* additionally supported the findings that the Zn-ion concentration for a given reaction time had a significant effect on the ZnO morphology.^46^ The presence of the dominant Zn(OH)_4_^2–^ species at later stages during the reactions was thereby argued to influence the heterogeneous secondary nucleation, similar to the observations seen in Figure 5a–c.^29^

Since the concentration of Zn-ionic species in our study was always the same, it is clear that the increased nucleation was caused by the presence of the CNF and triggered a larger consumption of Zn(OH)_4_^2–^ ions during the earlier stages of the reaction, closely following the addition of NaOH (aq). The consequence was that the saturation of the system decreased more rapidly at the beginning of the reaction, suppressing the occurrence of any dominant secondary nucleation. The presence of CNF can thus be used to prevent secondary growth processes, which occur as part of heterogeneous nucleation during the later stages of the reactions. The absence of formed secondary structures on the rod-based sea urchins suggested a limited nature of the more Zn-enriched surfaces at the sides of the rods to serve as nucleation sites.^21,22^

### The ZnO Morphology Development with Time

**Figure 6:**
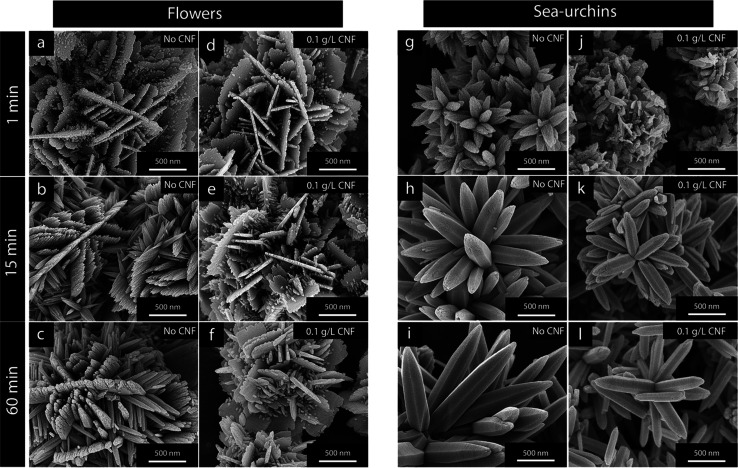
Flower-shaped ZnO particles obtained (a–c) in the absence of CNF and (c–e) with CNF present at 0.1 g/L for reaction times of (a, d) 1 min, (b, e) 15 min, and (c, f) 60 min. (g–l) The appearance of the sea urchin structures obtained (g–i) without the incorporation of CNF and (j–l) when incorporating 0.1 g/L of CNF at the same reaction times. The calcination was made at 400 °C with the purpose of highlighting the morphological ZnO differences after temperature exposure that solely removed the cellulose with unaffected ZnO structures.

Figure 6 shows calcinated samples of flower-shaped and sea-urchin-shaped ZnO structures obtained from aliquots extracted at different reaction times, 1, 15, and 60 min, with CNF absent or present during the synthesis, respectively.

For the flower-shaped particles in Figure 6a–f, the growth of secondary structures became visible after 15 min, with small spikes starting to appear on the surface of the sheets in Figure 6e, while the sheets in Figure 6b,c (60 min) do not display any small spikes on the primary sheet surfaces. Although the mechanism for the transformation from the sheet structures formed in the absence of CNF (1 min, Figure 6a) to sheets composed of associated rods after 60 min (Figure 6c) is unknown, it is clear that this transformation did not occur when 0.1 g/L of CNF was present in the solution (Figure 6d,f). It is suggested that this prevention of transformations during the progress of the 60 min reaction was caused by a more limited ability of the Zn-ion species to reform into novel structures as a consequence of the cellulose nanofiber network present in the solution.

The more enhanced nucleation and limited diffusion occurring when the CNF network was present in the solution also emphasized themselves in the early stages of the reactions generating sea urchin structures; see Figure 6g–l.

Figure 6g,j compares the 1 min aliquots and shows that the more dominant nucleation also was constant with more undefined morphologies with sizes of *ca.* 100–200 nm (Figure 6j). These increasingly mixed morphologies, as compared to the distinct rod-like structures shown in Figure 6g (when no CNF was present), were a consequence of a limited orientational growth where faceted edges did not develop.

It was also observed for both the 0.1 and 0.05 g/L reactions (not shown in Figure 6) that the total mass extracted after 1 min was *ca.* 30–50% smaller than that for the reference sample when no cellulose was present. In essence, 1 min after initiating the precipitation, the reactor containing no CNF allowed a more rapidly initiated growth and formation of larger 0.8 μm sea urchins, which in the long run resulted in strongly faceted structures, Figure 6h,i, with a smaller specific surface area and a smaller total yield of material. Accordingly, while the presence of CNF caused sheets with surfaces dominated by oxygen terminations to remain over time in the case of the flower-shaped particles, the CNF inclusion primarily had an impact on the ultimate size of the grown sea urchin structures with surfaces dominated by zinc terminations.^21,23^

### The Effect of CNF on Anisotropic ZnO Growth Characteristics

**Figure 7:**
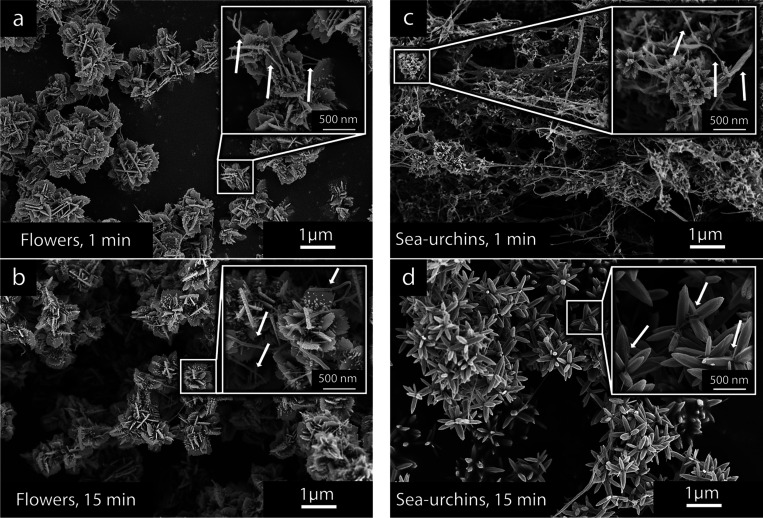
Micrographs showing the freeze-dried samples of the (a, b) flower-shaped and (c, d) sea-urchin-shaped ZnO structures made in the presence of CNF at a concentration of 0.1 g/L. The micrographs show the appearance of the freeze-dried ZnO/CNF material (a, c) 1 min and (b, d) 15 min into the reaction. The arrows highlight the presence of cellulose nanofibers in the extracted aliquots.

Figure 7 shows the freeze-dried material of the ZnO particles when mixed with the cellulose nanofibers (CNFs) after the reactions made in the presence of 0.1 g/L of CNFs. It was evident from the early 1 min extractions, compared to the extractions made after 15 min, that two different growth patterns could be elucidated for the flower-shaped particles and the sea-urchin-shaped particles. For the flower-shaped particles, the sheet morphology was already dominating the inorganic particles after 1 min (Figure 7a), and only minor amounts of CNF were visible due to the faster precipitation and greater amounts of formed ZnO. The presence of the sheet-consisting flower-shaped particles of almost identical sizes throughout the reaction (as observed in Figure 7b for the 15 min sample) confirmed that the zinc ionic species were mostly converted into solid ZnO within the first minute of the reaction. In contrast, as the first minute of the reaction progressed for the sea-urchin-shaped particles, the cellulose nanofibers remained the most prominent feature of the extracted sample (Figure 7c). The ZnO particles showed nanosized features scattered within the cellulose fiber network, which in size were consistent with the particles shown in Figure 6j.

After 15 min, the ZnO fraction of the material had obtained a more rod-like appearance, and the ratio between CNF and ZnO had decreased significantly. The observations were consistent with the fact that a significant amount of zinc ionic species remained dissolved in the reaction mixture for a longer time despite the presence of CNF. In previous research, it has been suggested that the Zn/OH ratio affects the formation of Zn(OH)_*x*_^*y*–^ species in the solution, ultimately influencing the morphologies of precipitated particles.^46−49^ Although this may also be applicable to the formation of either sheet-structured flower-shaped particles with dominant oxygen terminations^21^ or mostly zinc-terminated rod-structured sea urchin particles,^21^ it appears that the Zn(OH)_*x*_^*y*–^ species preceding the sheet formations required a lower level of saturation for the solid phase to form. In both cases, the cellulose network provided a strong nucleation support in the ZnO precipitation reaction, which was consistent with increased yields and smaller particles, although the CNF acted as a nucleation support at different extents and times for the two synthesis reactions. The reported phenomena and the ability of the CNF to act as a nucleation support may also have been influenced by the nonbasic character of the nitrate anions (associated with the metal salt used), showing limited interference with the metal ions associating with the cellulose.^50^

## Conclusions

The presence of cellulose nanofibers (CNFs) at ultra-low concentrations (0.1 g/L) increased the reaction yield with over 10% formed material of ZnO, regardless of specific ZnO morphologies, due to the CNF acting as a nucleation support. The average size of the ZnO sea-urchin-shaped particles decreased from *ca.* 2.45 to 1.34 μm (45%), while the diameter of the flower-shaped particles was reduced from *ca.* 3.5 to 1.8 μm (50%). The systemic decreases in particle sizes in combination with the increased yield led to an increase in the total surface area formed during the reaction, i.e., an increase in specific surface area shown to be *ca.* 60% for the flower-shaped and *ca.* 180% for the sea-urchin-shaped particles. The changes in morphologies as associated with the distinctly different particles with dominantly oxygen- or zinc-terminated surfaces were made possible to observe by adopting a methodology where ZnO particles were synthesized in the presence of cellulose, and the CNF subsequently was removed by calcination at 400 °C. This temperature was not sufficient for structural changes of the ZnO phase occurring at *T* > 500 °C.^21^ Overall, this study describes how cellulose nanofibers, as a crystalline biomacromolecule, can be used to systematically influence the morphologies of ZnO particles and subsequently their functional properties at low concentrations. The inorganic particle structures of an anisotropic nature will display different surface functionalities depending on exposed atomic surface lattices (*e.g*., photocatalytic activity), and cellulose can here be used to tune these properties. Cellulose is the most abundant crystalline natural polymer on earth and has been reported as highly versatile in its nature for various surface modifications, which opens up for new potential uses in applications involving metal oxide preparation.

## Supporting Information Available

Additional experimental details, materials and methods, and micrographs and experimental characterization (PDF)
