# Supplementary material for: Ultra-low Concentration of Cellulose Nanofibers (CNFs) for Enhanced Nucleation and Yield of ZnO Nanoparticles
Source: Langmuir. 2022 Oct 6;38(41):12480–90. doi: 10.1021/acs.langmuir.2c01713 (PMC9583615; doi:10.1021/acs.langmuir.2c01713)
Supplement: Supplementary file 1 — la2c01713_si_001.pdf [file la2c01713_si_001.pdf]

Supporting information

Ultra-low concentration of cellulose nanofibers (CNF) for enhanced nucleation and yield of ZnO-nanoparticles.

Billy W. Hoogendoorn[a], Björn K. Birdsong[a], Antonio J. Capezza[a] , Valter Ström[b], Yuanyuan Li[a], Xiong Xiao [a], and Richard T. Olsson*[a]

[a]Department of Fibre and Polymer Technology, KTH Royal Institute of Technology, Teknikringen 56, 11428 Stockholm, Sweden.
[b] Department of Material Science and Engineering, School of Industrial Engineering and Management, KTH Royal Institute of Technology, Brinellvägen 23, SE-100 44 Stockholm Sweden.

Corresponding authors.

E-mail addresses: *rols@kth.se (R. Olsson)

Table **S**1. The specifications in terms of reaction temperature and the concentrations of the Zinc-salt solutions, CNF-dispersions and sodium hydroxide solutions for the additional reactions (referred to as Run 7-8) done for the flower-shaped particles at higher CNF-concentrations.

Sample order	Sample specification	$C_{Zn(NO_3)_2 6(H_2O)}$ [M]	$C_{CNF\text{-}disp}$ [g/L]	C_{NaOH} [M]	C_{CNF} [g/L]*	T (°C)
Run 7	ZnO_{CNF3}-Flower	0.45	3	3	1.5	40
Run 8	ZnO_{CNF4}-Flower	0.45	3.6	3	1.8	40

Table **S2.** XRD-data of freeze-dried ZnO-samples obtained for flower-shaped ZnO-particles synthesized at CNF concentrations of 1.5 g/L and 1.8 g/L.

Sample order	Sample specification	Crystallite size (nm)*	Peak intensity ratio (002)/(101)
Run 7	ZnO_{CNF3}-Flower	26.0	0.67
Run 8	ZnO_{CNF4}-Flower	30.0	0.72

*The crystallite size was determined by using the Scherrer equation on the (002)-peak.

Table S3. Zn-concentrations in the aqueous supernatant after being stirred for 60 min at 60°C with and without the presence of CNF as measured with ICP-measurements.

Sample	C_{CNF} (g/L)	$C(Zn^{2+})$		
		C_{Ref} (mg/Kg)	$C_{BC\text{-}supernatant}$ (mg/Kg)	$C_{BC\text{-}material}$* (mg/Kg)
$Zn(NO_3)_2(H2O)_6$ + CNF (aq)	3	7200	7120	8000

The BC-material collected after centrifugation had an estimated solid content of ca. 3%

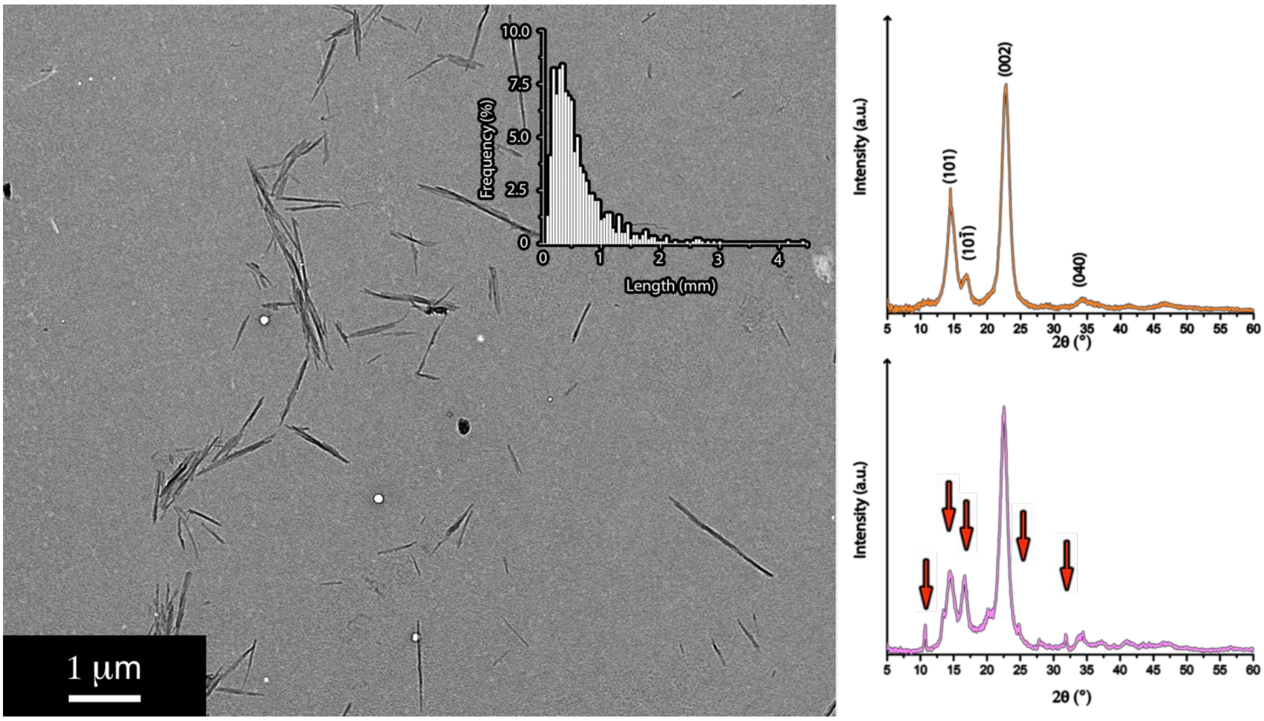

Fig. S1. TEM-Micrographs cellulose nanofibers obtained after acid hydrolysis. The histograms reveal the length and thickness distributions of the CNF. The XRD-diffractograms to the right reveal the diffraction patterns of freeze-dried bacterial CNF (top right) and freeze-dried bacterial CNF after being mixed with $Zn(NO_3)_2(H_2O)_6$ for three hours at 60°C (see experimental for further information). The arrows highlight the additional peaks occurring due to the formation of a $Zn(NO_3)(OH)$-phase.

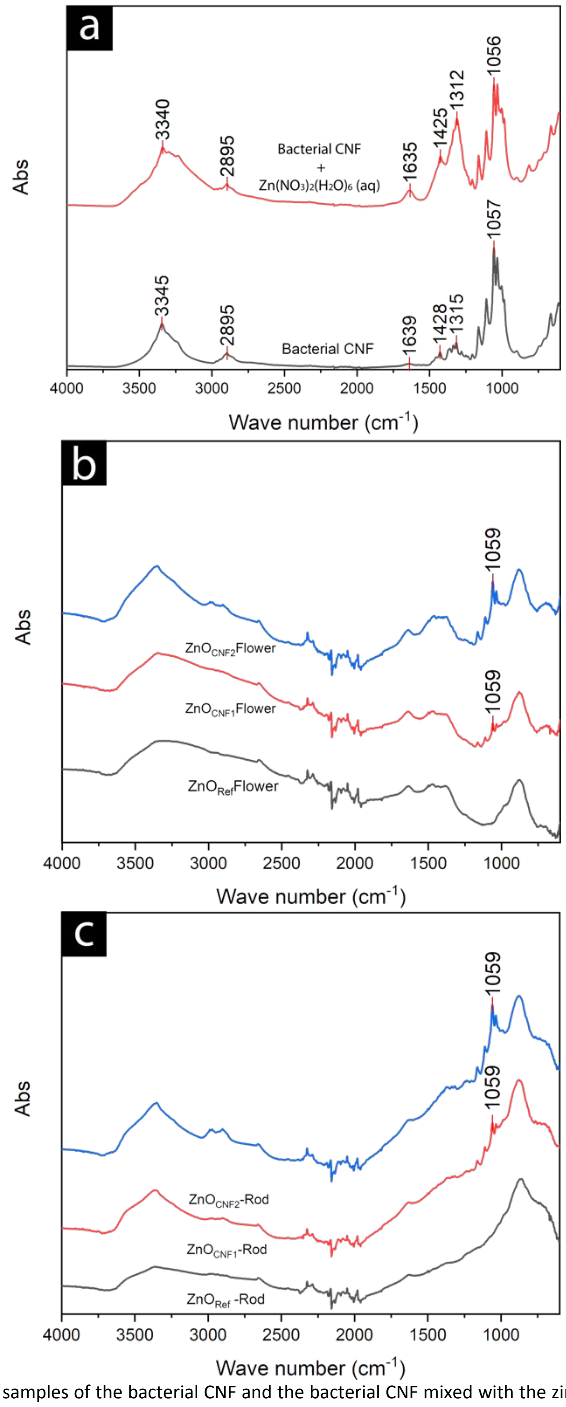

Fig S2. (a) FTIR-spectra of freeze-dried samples of the bacterial CNF and the bacterial CNF mixed with the zinc nitrate hexahydrate according to the concentrations prior to the initiation of the synthesis. The highlighted peaks at wavelengths 3340-3345 and 2895 cm^{-1} represents the stretching of the OH-groups and CH-groups respectively. The peaks at 1635-1639 cm^{-1} represents the stretching of the OH-groups from the absorbed water. Additionally, the peak at ca. 1430 represents C-H and O-CH-stretching while the peak at ca. 1310 represents the bending of C-H-bond. (b) The resulting material from the reactions producing ZnO-flowers (b) and ZnO-sea-urchins (C). The signals highlighted peaks at 1059 cm^{-1} represents the stretching of the C-O-C associated with cellulose.

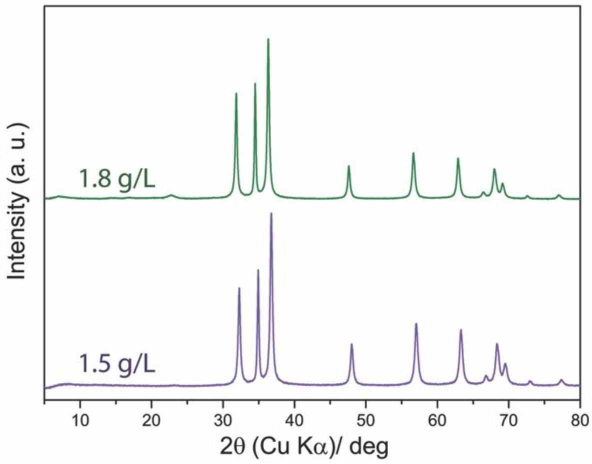

Fig. S3. XRD-diffractogram of freeze-dried samples obtained from the reactions producing flower-shaped particles at CNF-concentrations of 1.5 g/L and 1.8 g/L

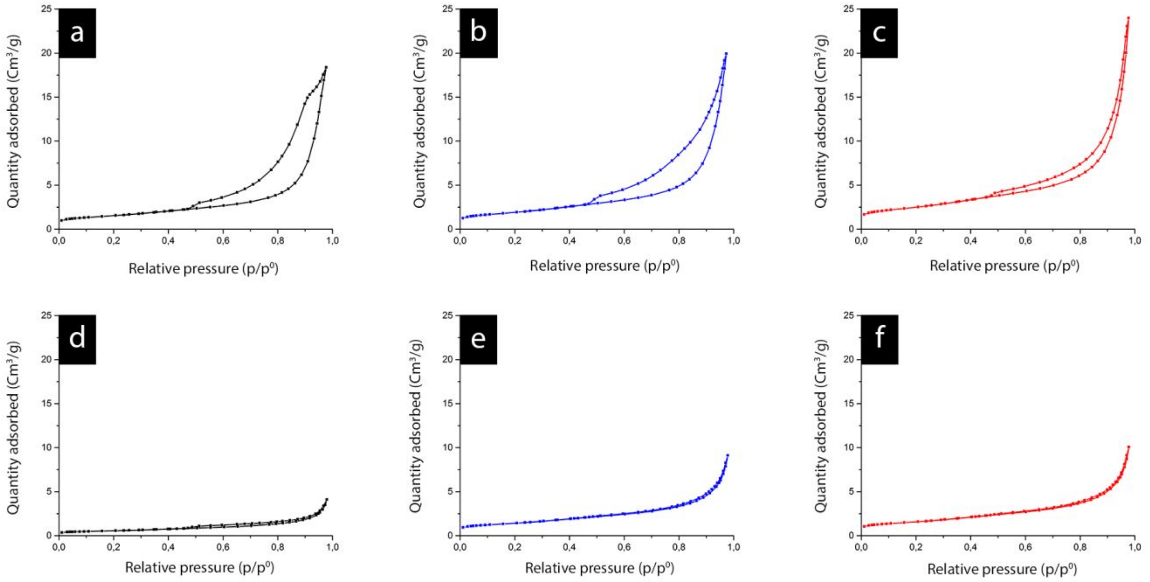

Fig. S4. BET-isotherms obtained for the flower-shaped particles obtained at CNF-concentrations of 0 g/L (a), 0.05 g/L (b) and 0.1 g/L (c) as well as for the sea-urchin-shaped particles obtained at 0 (d),), 0.05 g/L (e) and 0.1 g/L (f).

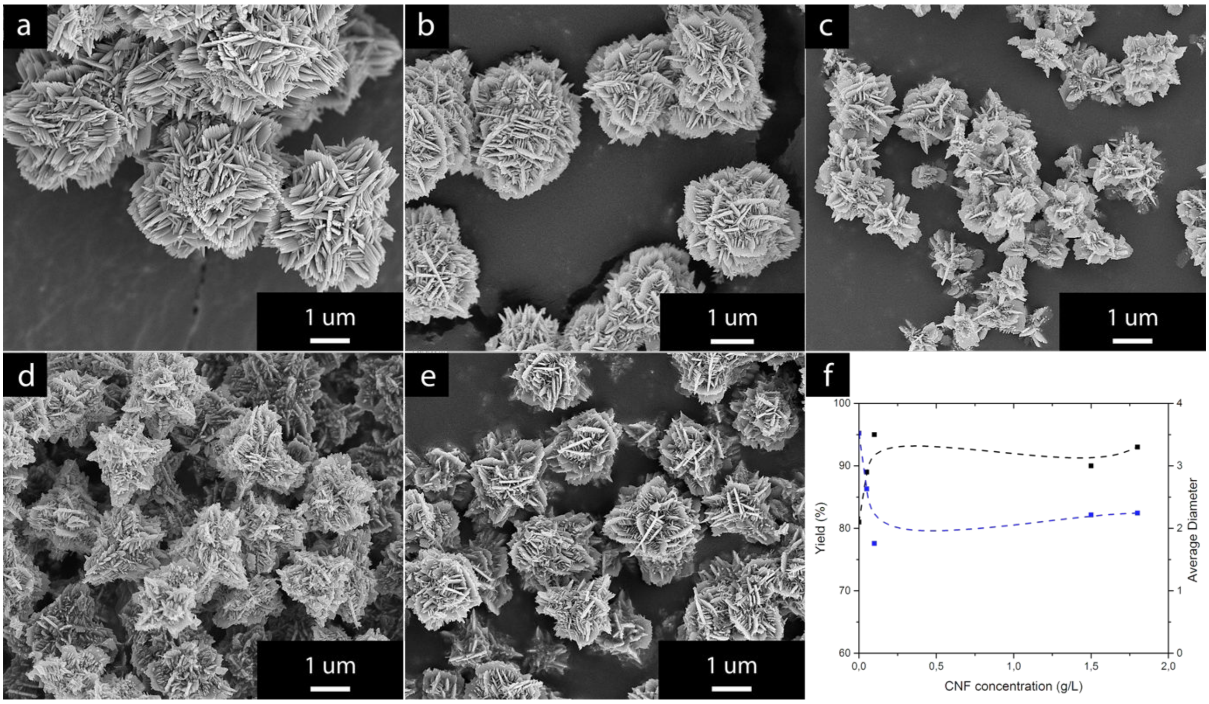

Fig. S5. SEM-Micrographs of heat-treated ZnO-particles obtained for the flower-shaped particles at CNF-concentrations of (a), 0 g/L (b), 0.05 g/L (c), 0.1 g/L (d), 1.5 g/L (e) and 1.8 g/L after the addition of NaOH (aq). (f) Average diameter of ZnO-particles obtained through ImageJ (blue dots) and reaction yield (black dots) in relation to CNF-concentration in the ZnO-reaction after initiation. The dotted lines are used as a rough estimation of the occurring trends when increasing the CNF-concentration.
